# Diagnostic Accuracy of a Blood-Based Biomarker Panel for Colorectal Cancer Detection: A Pilot Study †

**DOI:** 10.3390/cancers16244176

**Published:** 2024-12-15

**Authors:** Elba V. Caraballo, Hilmaris Centeno-Girona, Brenda Carolina Torres-Velásquez, Madeline M. Martir-Ocasio, María González-Pons, Sheila N. López-Acevedo, Marcia Cruz-Correa

**Affiliations:** 1 Division of Clinical and Translational Cancer Research, University of Puerto Rico Comprehensive Cancer Center, San Juan 00921, Puerto Rico; hcenteno@cccupr.org (H.C.-G.); btorres@cccupr.org (B.C.T.-V.); mmartir@cccupr.org (M.M.M.-O.); maria.gonzalez9@upr.edu (M.G.-P.); shelopez@cccupr.org (S.N.L.-A.); marcia.cruzcorrea@upr.edu (M.C.-C.); 2 School of Medicine, Medical Sciences Campus, University of Puerto Rico, San Juan 00921, Puerto Rico

**Keywords:** blood-based biomarkers, diagnostic accuracy, colorectal cancer, Hispanics

## Abstract

**Background:** Colorectal cancer (CRC) is a leading cause of death worldwide. Despite its preventability through screening, compliance still needs to improve due to the invasiveness of current tools. There is a growing demand for validated molecular biomarker panels for minimally invasive blood-based CRC screening. This study assessed the diagnostic accuracy of four promising blood-based CRC biomarkers, individually and in combination. **Methods:** This case–control study involved plasma samples from 124 CRC cases and 124 age- and sex-matched controls. Biomarkers tested included methylated DNA encoding the Septin-9 gene (*mSEPT9*) using Epi proColon^®^ 2.0 CE, insulin-like growth factor binding protein 2 (IGFBP2), dickkopf-3 (DKK3), and pyruvate kinase M2 (PKM2) by ELISA. Diagnostic accuracy was measured using the receiver operating characteristic (ROC), area under the curve (AUC), as well as sensitivity and specificity. **Results:** Diagnostic accuracy for *mSEPT9*, IGFBP2, DKK3, and PKM2 was 62.9% (95% CI: 56.8–62.9%), 69.7% (95% CI: 63.1–69.7%), 61.6% (95% CI: 54.6–61.6%), and 50.8% (95% CI: 43.4–50.8%), respectively. The combined biomarkers yielded an AUC of 74.4% (95% CI: 68.1–80.6%), outperforming all biomarkers except IGFBP2. **Conclusions:** These biomarkers show potential for developing a minimally invasive CRC detection tool as an alternative to existing approaches, potentially increasing adherence, early detection, and survivorship.

## Simple Summary

This study investigates four blood-based biomarkers, *mSEPT9*, IGFBP2, DKK3, and PKM2, for colorectal cancer (CRC) detection. Current CRC screening methods are often invasive, leading to low compliance. We evaluated the diagnostic accuracy of these plasma biomarkers individually and their combinations in 124 CRC patients and 124 healthy controls. Our results showed that the combination of plasma biomarkers improved accuracy; the combined model outperformed individual biomarkers in detecting CRC. These findings suggest that a multi-biomarker blood test could offer a less invasive, effective alternative for CRC detection, potentially improving early detection rates and patient adherence to screening guidelines. Further research is needed to validate these findings in larger, diverse populations and explore their integration into routine clinical practice.

## 1. Introduction

Colorectal cancer (CRC) is the second cause of cancer-related deaths globally among men and women [1]. In 2022, 1,926,425 million new CRC cases and 904,019 deaths were estimated, accounting for 9.3% of all cancer deaths [2]. Overall, in the United States (US), CRC is the second cause of cancer-related deaths and the first leading cause for Puerto Rican Hispanics (PRHs) [3,4]. PRHs are a diverse population arising from a genetic admixture of African, European, and Native American ancestries [5]. This genetic background could influence cancer susceptibility, tumor characteristics, and the effectiveness of screening methods [6]. According to the Puerto Rico Central Cancer Registry (PRCCR), from 2016 to 2020, CRC was the second most diagnosed cancer in PRHs by sex, accounting for 11.5% of all cancer cases in men and 10.5% of all cancers in women [4]. This underscores the importance of improving screening methods to enhance compliance and early detection rates in PRHs.

The Centers for Disease Control estimates that 68% of CRC-related deaths could be prevented through adherence to screening [7]. Current guidelines recommend using stool-based tests or imaging studies to screen for CRC in adults aged 45 to 75 [8]. Imaging studies, such as colonoscopies or computed tomography (CT) colonography, allow for early detection of CRC and reduction in mortality [8] but are associated with higher costs [9] and carry the risk of bleeding and perforation due to their invasive nature [8]. In contrast, stool-based screening tests are relatively non-invasive but have low specificity for CRC [10,11]. Despite the high specificity (91%) and sensitivity (94%) reported for the stool DNA test Cologuard and next-generation multitarget stool DNA (sensitivity: 93.9% and specificity: 90.6%), these have not been validated in Hispanic populations [10,12]. Several barriers for the implementation of colonoscopy and stool-based CRC screening methods include cost, bowel preparation, and reluctance to submit stool samples, factors likely influenced by behavioral, cultural, and socioeconomic factors [13,14,15]. CRC screening rates in Puerto Rico are lower than in the mainland United States [16,17], and compliance with screening guidelines among age-eligible individuals living in Puerto Rico is only about 56% [18], or roughly 12% lower than the Healthy People 2030 target goal for colorectal cancer screening [19].

There is a growing demand for blood-based tests to detect CRC, particularly among populations at higher risk for developing cancer and those with lower screening rates [20]. Given the minimally invasive nature of blood-based tests and their widespread use in overall health monitoring, these tests are crucial for improving screening compliance for CRC. An example is the recently approved cell-free DNA (cfDNA) Guardant test. The cfDNA is a blood-based test that allows detection and monitoring of the disease by identifying tumor-derived DNA fragments circulating in the bloodstream [21]. Evidence shows that the uptake of blood-based screening strategies is approximately 11% higher than stool-based tests, suggesting a more effective alternative for CRC screening [22]. However, further work is needed to develop a blood-based CRC screening approach with strong diagnostic accuracy in diverse populations.

Several biomarkers have emerged as potential candidates for the blood-based detection of CRC [21,23,24,25,26,27,28]. Among the most promising are the circulating DNA biomarker methylated Septin-9 (m*SEPT9*) and serum proteins insulin-like growth factor binding protein 2 (IGFBP2), dickkopf-3 (DKK3), and pyruvate kinase M2 (PKM2) [29,30,31,32]. The FDA has approved blood-based *mSEPT9* testing as a minimally invasive limited alternative to standard stool- or imaging-based CRC screening. The protein biomarkers IGFBP2, DKK3, and PKM2 were previously identified and validated in an Australian cohort for their diagnostic potential, outperforming stool-based screening tests for detecting early stages of CRC [33].

In this study, we evaluated the diagnostic accuracy of these four promising biomarkers individually and as a biomarker panel for detecting CRC among PRHs. To our knowledge, this is the first case–control study designed to assess the sensitivity and specificity of these blood biomarkers, alone and in combination, in our population. We hypothesized that the diagnostic accuracy of the integrated panel would outperform the individual biomarkers, overall and in a stage-dependent manner. This work represents a first step in the development of clinical immunodiagnostic tests that have the potential to improve adherence to CRC screening and ultimately increase survival [34].

## 2. Materials and Methods

### 2.1. Study Population and Study Design

A case–control study design was used to evaluate the diagnostic accuracy of plasma m*SEPT9*, IGFBP2, DKK3, and PKM2 in a cohort of 248 PRHs. Plasma samples of 124 cases with CRC and 124 age- (within ±5 years) and sex-matched controls (healthy individuals) aged 40–75 years were obtained from the Puerto Rico Familial Colorectal Cancer Registry (PURIFICAR) [35,36]. PURIFICAR is an island-wide population-based registry that recruits and collects biospecimens (blood, colorectal tissue, and stool) from both cases (individuals with colorectal neoplasia) and controls (healthy individuals without prior history of colorectal neoplasia) in six affiliated locations. For the CRC group, only plasma samples from individuals with pathology-confirmed CRC and no history of Lynch Syndrome were selected; meanwhile, the control group comprised plasma samples from healthy individuals with negative colonoscopies and no history of colorectal adenomas or CRC. Individuals with hereditary cancer syndromes and inflammatory bowel disease were excluded. The PURIFICAR research protocol was approved by the University of Puerto Rico Medical Sciences Campus Biosafety Committee and Institutional Review Board (IRB # A2210207/2290034759).

### 2.2. Data and Biospecimen Collection

#### 2.2.1. Demographic and Clinicopathological Data

Demographic data, including age, sex (biological attribute), education and marital status, dietary and lifestyle habits, health insurance, geographic region, co-existing metabolic comorbidities, and family history of cancer, were obtained through PURIFICAR. Clinicopathological variables, including primary tumor location, categorical tumor stage (I, II, III, IV), and genetic ancestry [6], were also obtained from the registry. The tumor stage variables were dichotomized into early (I/II) and advanced stages (III/IV) of the disease.

#### 2.2.2. Biospecimen Collection

During recruitment, blood samples were drawn from CRC patients and healthy individuals. The samples were collected in EDTA-plasma tubes and processed to obtain plasma and peripheral blood lymphocytes (PBMCs) [37]. Samples were stored at −80 °C until used in further studies.

### 2.3. Laboratory Methods

#### 2.3.1. Detection of Serum DNA Methylation Biomarker *mSEPT9*

Plasma *mSEPT9* levels were measured using the Epi proColon^®^ 2.0 CE test by Polymedco Inc. (Cortlandt Manor, NY, USA), following the manufacturer’s instructions (M5-02-001, M5-02-002, M5-02-003). The Epi proColon test involves two primary procedural phases. First, 3.5 mL of plasma from cases or controls were mixed with an equal amount of lysis buffer and incubated for 10 min. After incubation, freshly suspended magnetic beads and absolute ethanol were added, and samples were rotated for 45 min. Subsequently, magnetic beads were washed, and DNA was eluted. Next, purified DNA was incubated with bisulfite solution for 45 min at 80 °C to produce bisulfite-converted DNA (bisDNA). Second, bisDNA was re-purified using freshly suspended magnetic beads and assayed by a duplex Real-Time PCR (Applied Biosystems^®^ 7500, Applied Biosystems, Waltham, MA, USA) with simultaneous *mSEPT9* and ACTB (β-actin) detection. Samples were classified as either *mSEPT9* positive or negative based on cycle threshold (Ct) values, per the manufacturer’s recommendations.

#### 2.3.2. Detection of Serum Protein Biomarkers: IGFBP2, DKK3, and PKM2

Plasma levels of IGFBP2 (Assay Solution Catalog #: AYQ-E10773), DKK3 (Assay Solution Catalog #: AYQ-E10862), and PKM2 (Biorbyt Catalog #: orb441606) were tested using commercially available ELISA kits following the manufacturer’s instructions (Assay Solutions, Woburn MA and Biorbyt, Cambridge, UK) by Novatein Biosciences, Inc. Plates were read at 450 nm using a microplate reader (Molecular Device SpectraMAX GeminiEM). For each assay, samples and working standards were tested in duplicate and an average concentration of the biomarkers was used for statistical analysis (Assay Solutions, Woburn, MA, USA and Biorbyt, Cambridge, UK). Biomarker concentrations (pg/mL) were calculated from their respective standard curves using SoftMax Pro software 7.1.

#### 2.3.3. Ancestry Informative Markers (AIMS) Panel Genotyping

A total of 105 AIMs panels were genotyped using PBL genomic DNA on the Sequenom MassArray iPLEX technology (Sequenom, San Diego, CA, USA), as described in Perez-Mayoral et al., 2019 [6]. This AIMs panel has been validated for calculating continental ancestry information in admixed Latino groups, including Puerto Ricans. It includes of SNP markers that inform European, African, and Amerindian ancestry. For genotype SNP calls, Sequenom TYPER 4.0 software (Sequenom, San Diego, CA, USA) was used. A model-based clustering technique was employed to determine each participant’s individual ancestry estimations using the STRUCTURE v2.3 program (Stanford, CA, USA).

### 2.4. Statistical Analysis

#### 2.4.1. Study Power and Sample Size Calculation

Previous studies have reported area under the receiver operating curve (ROC-AUC) values ranging from 0.60 for one biomarker (IGFBP2) to 0.93 for a three-biomarker model composed of DKK3, PKM2, and IGFBP2 [33]. AUC values for *mSEPT9* have also been reported within this range [38]. In the current study, it was calculated that for an acceptable biomarker specificity limit of 0.60 with an alpha of 0.05 and a case–control ratio of 1:1, a total of 248 samples (124 CRC cases and 124 healthy controls) would be needed to reach a statistical power of 90%. The sample size estimation was performed using the one ROC curve with a given AUC test (two-sided) of the R package pROC v. 1.18.5 [39].

#### 2.4.2. Data Processing

Optimal cut-offs for IGFBF2, DKK3, and PKM2 biomarkers were calculated to transform continuous values to binary using different methods: Median, Youden index (Youden), simultaneous maximum sensitivity and specificity (MaxSpSe), equality of sensitivity and specificity (SpEqualSe), maximize efficiency and accuracy (MaxEfficiency), maximizing sensitivity (MaxSe), maximizing specificity (MaxSp), and minimizing *p*-value associated with the test (MinPvalue). The median value of each biomarker was selected to perform the transformation, and biomarkers with transformed values are referred to throughout the manuscript as binary biomarkers. Cut-offs were computed using the OptimalCutpoints v. 1.1-5 R Package [40].

#### 2.4.3. Descriptive Analysis

Descriptive statistics were calculated to summarize demographic and clinicopathological characteristics. Pearson chi-squared tests were used to compare differences in demographic and clinicopathological variables, as well as *mSEPT9* positivity. The Mann–Whitney–Wilcoxon test for median values was used to test differences between cases and controls for IGFBP2, DKK3, and PKM2 biomarkers, overall and by tumor stage (early or advanced). Bonferroni correction was used to account for simultaneous multiple comparisons. Significance was set at α = 0.05 for all analyses.

#### 2.4.4. Univariate Analysis

Simple binomial generalized linear models (GLMs) with log link were used to create a single model for each biomarker, with biomarkers using their continuous values as predictors of CRC. The AUC-ROC was estimated to evaluate the diagnostic accuracy of each biomarker. The AUC-ROC and corresponding 95% confidence intervals (CIs) were calculated from these models, overall and by stage. Sensitivity and specificity with a corresponding 95% CI were computed using binary biomarkers, also overall and by stage.

#### 2.4.5. Multivariate Analysis

Multivariate binomial GLMs with log link were developed using combinations of biomarkers to evaluate their predictive values for CRC. IGFBP2, DKK3, and PKM2 biomarkers were included as continuous values. The multivariate models were defined as follows: Model 1 included all four biomarkers (*mSEPT9*, IGFBP2, DKK3, and PKM2); Model 2 included only *mSEPT9*, IGFBP2, and DKK3; and Model 3 included only *mSEPT9* and IGFBP2.

After running multivariate models, a SpEqualSe cut-off was used to classify the fitted values (continuous nature) into binary values (binary fitted values). If the fitted value was equal to or greater than the threshold, a positive predicted case of CRC was assumed. Otherwise, it was considered a negative predicted case. Next, a confusion matrix was created with the binary fitted values, CRC diagnosis, and true outcome to compute sensitivity, specificity, and AUC-ROC with the corresponding 95% Cis, overall and by stage.

The nonparametric DeLong test [41] was used to compare ROC curves to determine whether there were differences in the AUC in pairwise comparisons among univariate and multivariate models.

#### 2.4.6. Statistical Software

Data were analyzed using R-Studio, an Integrated Development Environment for R (R-Studio, Inc.), with the packages “pwr”, “pROC”, and “binom” from R software (V.3.6.3, R Foundation for Statistical Computing, Vienna, Australia) and R-Studio Integrated Development Environment for R.

## 3. Results

### 3.1. Demographic Characteristics

The demographic features of the study cohort are summarized in Table 1. The median age of study participants was 58 (33–85) years among CRC cases and 55 (26–83) years among healthy controls (*p*-value = 0.120). Males accounted for 50.8% of the study population. CRC cases did not differ from healthy controls regarding educational attainment, marital status, body mass index, insurance coverage, geographic health region, lifestyle (smoking or alcohol intake status) factors, comorbid diabetes mellitus, or family history of CRC (*p*-values > 0.05).

### 3.2. Clinicopathological and Molecular Characteristics

Table 1 summarizes the clinicopathological characteristics of the CRC cases and controls in this study. Most CRC cases were classified as early-stage disease (52.0%, *n* = 65) and located in the distal colon (79.1%, *n* = 91). Genetic admixture analysis did not reveal associations between African, Amerindian, or European genetic ancestry and CRC (*p*-values > 0.05).

### 3.3. Individual Biomarker Analysis

We measured *mSEPT9*, IGFBP2, DKK3, and PKM2 plasma levels in samples from CRC cases and healthy PRHs and stratified our findings by stage (Table 2). In our overall analysis, three individual biomarkers were elevated in CRC cases compared with the controls: *mSEPT9* (*p* < 0.001), IGFBP2 (*p* < 0.001), and DKK3 (*p*-value = 0.002). PKM2 levels did not differ between CRC cases and controls in our cohort (*p*-value = 0.834). When stratified by stage, these patterns remained. In early- stage CRC, *mSEPT9*, IGFBP2, and DKK3 levels reached significance (*p*-value = 0.002, *p*-value = 0.004, and *p*-value = 0.032, respectively), but not PKM2 (*p*-value = 0.320). Similar findings were observed in advanced-stage CRC cases (*p*-value = 0.012, *p* < 0.001, *p*-value = 0.026, *p*- value = 0.138, respectively). Moreover, the results presented in Table 3 show that *mSEPT9*, IGFBP2, and DKK3 levels did not significantly differ between early and advanced stages of CRC (*p*-values > 0.05). Meanwhile, PKM2 levels were significantly higher in the early stages when compared with the advanced stages (*p* = 0.0014).

**Figure 1:**
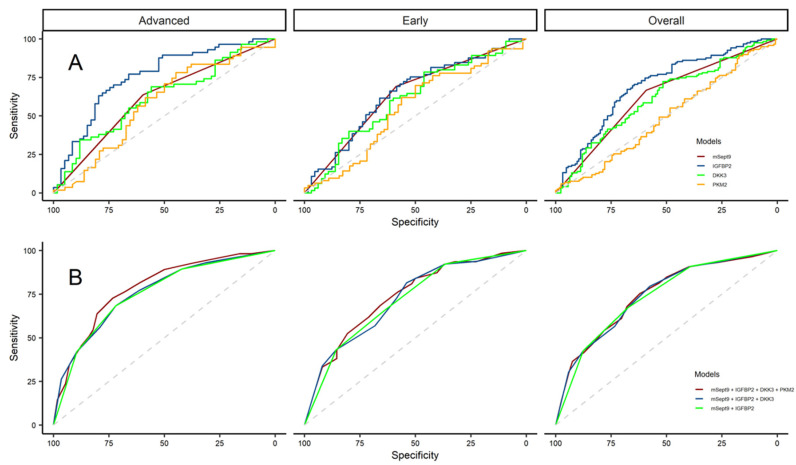
Evaluation of biomarker diagnostic accuracy for CRC. (**A**) ROC plots of *mSEPT9*, IGFBP2, DKK3, and PKM2 analyzed individually for CRC detection in the overall cohort (left panel) or stratified by tumor stage: early-stage disease (middle panel) or advanced-stage disease (right panel). Red = *mSEPT9*, blue = IGFBP2, green = DKK3, and gold = PKM2. (**B**) ROC plots of *mSEPT9*, IGFBP2, DKK3 and PKM2 analyzed with multivariate models for CRC detection in the overall cohort (left panel), early-stage disease (middle panel), or advanced-stage disease (right panel). Analyses included all four putative biomarkers (Model 1, red), *mSEPT9* + IGFBP2 + DKK3 (Model 2, blue), and *mSEPT9* + IGFBP2 (Model 3, green).

The diagnostic performance of the individual biomarkers was evaluated using ROC curves (Figure 1) and AUC analyses (Table 4). IGFBP2 demonstrated the best performance as an individual biomarker. Its performance was strongest in advanced-stage disease (AUC 75.5%, 95% CI 66.6–84.4) compared with early-stage disease or the overall cohort (AUC 64.6%, 95% CI 55.0–74.1 and AUC 69.7%, 95% CI 63.1–76.3, respectively). IGFBP2 also yielded higher AUC, sensitivity, and specificity values across stages compared with the other biomarkers studied (Table 3), except for early-stage disease, where *mSEPT9* had higher performance (*mSEPT9*: 69.2, 95% CI 56.6–80.0 vs. IGFBP2: 66.2%, 95% CI 53.4–77.4).

### 3.4. Multivariate Analysis

GLM and derived analyses were used to evaluate whether the use of the combined biomarkers in a multivariate model would maximize their accuracy in distinguishing CRC from the controls. ROCs for this approach are presented in Figure 1 and the diagnostic performance of these models are summarized in Table 5. We observed comparable AUC results across models, with Model 1 (combination of four biomarkers) yielding the best performance overall and by stage. The highest AUC for any model in this study was with Model 1 in advanced-stage disease (75.4%, 95% CI 66.7–84.0). Model 1 had higher sensitivity compared with Models 2 and 3 in the overall cohort and early-stage disease (Table 4). However, in advanced-stage disease, Models 2 and 3 outperformed Model 1 regarding sensitivity. In the overall analysis and among cases with early-stage disease, we observed the highest specificity with Model 3, while Model 1 yielded the highest specificity for advanced-stage disease (Table 5). The DeLong test indicated no significant differences in the AUC between the multivariate models, but significant differences were found between Model 1 and univariate analyses of *mSEPT9*, DKK3, or PKM2 (*p* < 0.05, Table 6).

## 4. Discussion

This study evaluated the diagnostic accuracy of *mSEPT9*, IGFBP2, DKK3, and PKM2 as blood-based biomarkers for CRC detection. Our findings showed that the plasma levels of individual biomarkers *mSEPT9*, IGFBP2, and DKK3 significantly differed between CRC cases and healthy controls for both early and advanced stages of CRC. The combination of *mSEPT9*, IGFBP2, DKK3, and PKM2 as a panel (Model 1) improved the diagnostic accuracy for CRC detection compared with the individual biomarkers.

The sensitivity of m*SEPT9* in our study population aligns with the range reported in other studies, although its specificity was notably lower [42,43]. *mSEPT9* may have shown higher sensitivity in early stages due to its involvement in early carcinogenesis processes, where epigenetic changes such as DNA methylation can be more readily detected. Additionally, lower levels of DNA methylation have been reported in the Hispanic population, as observed in previous studies [44,45].

IGFBP2 was the best-performing individual biomarker, consistent with previous reports of elevated serum levels in various malignancies, including CRC [46,47,48]. IGFBP2’s role in the Wnt/β-catenin signaling pathway is well documented. Elevated levels in CRC patients may result from interactions with the L1/ezrin/NF-κB pathway, promoting cell motility and tumorigenesis [49]. Additionally, IGFBP2 facilitates IGF signaling, which is crucial for cancer cell proliferation and survival [50,51,52].

Moreover, DKK3 findings were consistent with the previous literature [53] but showed a lower AUC than others [54,55]. DKK3 belongs to a family of secretory *Wnt* signaling modulators that suppress cell growth, differentiation, and proliferation [56,57,58]. The variability in DKK3 levels across different populations could be due to differences in tumor microenvironments, which affect its production and utilization. The role of DKK3 in modulating *Wnt* signaling and its suppression of cell growth and differentiation indicate its complex involvement in CRC pathogenesis. Although PKM2 is crucial for catalyzing the final step of glycolysis and linking nuclear responses to nutrient availability, we did not find significant differences in PKM2 levels between the CRC cases and controls [33,59,60]. This discrepancy might be due to population-specific genetic and environmental factors.

Furthermore, our results suggest that while there were no differences among the AUC of the multivariate models, Model 1 performance was superior to Models 2 and 3 based on sensitivity (overall and early-stage disease) and specificity (advanced-stage disease) measures under the specified conditions. Overall, our panel showed similar sensitivity and lower specificity than those observed by Fung and colleagues (sensitivity: 73%, specificity: 95%). Further work is needed to compare the efficacy of this model to established screening tests such as the fecal occult blood test (FOBT) (pooled sensitivity: 31% and specificity: 87%) [61], fecal immunochemical test (FIT) (sensitivity: 75.0% and specificity: 90.1%) [62], and the next-generation multitarget stool DNA test (sensitivity: 93.9% and specificity: 90.6%) [12]. As hypothesized, increased diagnostic accuracy for CRC was observed using the biomarker panel compared to each blood-based biomarker individually. Additionally, this model performed better among advanced stages with a sensitivity of 63.6% (95% CI, 49.6–76.2) and specificity of 80.4% (95% CI, 67.61–89.8).

Recent advances in blood-based tests, such as the cfDNA, have shown promise in CRC screening due to their non-invasive nature and high sensitivity and specificity. While the cfDNA Guardant test has proven effective (sensitivity: 83.1% and specificity: 89.6%) [21], similar to our biomarker panel, our study offers additional advantages by integrating multiple biomarkers interacting in different biological pathways associated with CRC. Our full model shows an acceptable diagnostic accuracy for both early and advanced CRC, suggesting that the proposed panel of blood-based biomarkers could be a promising alternative for detection. A combined panel including cfDNA and other biomarkers in the biological pathway may provide additional diagnostic accuracy and should be developed across diverse populations.

The integration of multi-biomarker blood-based tests into routine CRC screening guidelines is critical to address significant barriers associated with compliance with traditional methods. Unlike a colonoscopy, which often requires extensive preparation, travel, and time off work, non-invasive blood tests can be administrated during routine healthcare visits. This practicality makes them suitable for underserved populations including those in rural or low-income areas, where logistical barriers to screening are more prevalent [63,64]. Furthermore, the cost of blood-based tests can range from USD 192 (e.g., Epi proColon) to USD 949 (e.g., liquid biopsy), which is significantly lower than the USD 1100 to USD 1700 cost of a colonoscopy, depending on whether a polypectomy is performed [65,66,67]. Although blood tests do not allow for immediate therapeutic interventions, their affordability and non-invasive nature offer a strategy to improve adherence to screening. Additionally, blood tests have been shown to reduce CRC incidence by 40% and mortality by 52% compared with no screening, with a cost per quality-adjusted life-year of USD 25,600–USD 43,700, making them cost-effective in populations with lower adherence to other cancer screening methods [66]. Their ability to detect advanced precancerous lesions and early-stage CRC highlights the utility of our findings for population-wide screening. An adequate multi-biomarker panel could achieve parity or surpass the cost-effectiveness of existing modalities while improving accessibility, enhancing compliance and addressing health inequities.

Our study provides valuable insights into the diagnostic accuracy of m*SEPT9*, IGFBP2, DKK3, and PKM2 in CRC; however, several limitations should be acknowledged. First, given that the study sample size was relatively small, a validation in larger, population-based samples is necessary to confirm the diagnostic utility of the proposed biomarker panel. Although strict protocols were followed for technical aspects, differences in blood sample collection, processing, and storage could lead to variability in biomarker levels and diagnostic performance. Moreover, different methodological platforms can lead to varying results and higher rates of false positives. Hence, studies using integrated methodologies such as multiplexed ELISA or epigenomics assays are warranted to ensure data reproducibility and consistency. Also, considering our data, prospective studies are needed to evaluate our blood-based biomarker panel’s performance characteristics (AUC-ROC, sensitivity, and specificity) combined with existing risk prediction models among Hispanic subpopulations [68,69]. Additionally, incorporating machine learning techniques, such as random forests, extreme gradient boost, or support vector machines [70], will enhance the predictive accuracy and clinical utility of this blood-based multi-biomarker panel.

## 5. Conclusions

To our knowledge, this is the first case–control study to assess the sensitivity and specificity of these blood-based biomarkers in PRHs. Our findings suggest that combining a panel of selected biomarkers improves diagnostic accuracy for CRC detection, with IGFBP2 and *mSEPT9* performing similarly as individual biomarkers in early and advanced stages. This study contributes to the ongoing efforts to identify effective, minimally invasive diagnostic tools capable of accurately detecting CRC at an early stage through a panel of biomarkers. Future studies should explore these biomarkers’ underlying biological pathways and clinical significance. This work represents a significant step toward improving CRC detection and outcomes across different populations.

## Figures and Tables

**Table 1:** Clinicopathological characteristics among CRC cases and healthy controls.

Characteristics	Control (*n* = 124)	CRC (*n* = 124)	*p*-Value
**Median age [min–max] ^a^**	54.5 [26.0–83.0]	58.0 [33.0–85.0]	0.120
**Age group ^b^**			
<50	39 (31.5)	36 (29.0)	0.782
≥50	85 (68.5)	88 (71.0)	
**Gender ^b^**			
Female	61 (49.2)	61 (49.2)	0.999
Male	63 (50.8)	63 (50.8)	
**Educational attainment ^b^**			
<12	48 (47.5)	48 (51.1)	0.726
≥12	53 (52.5)	46 (48.9)	
**Marital status** ^b^			
Single/divorced/widowed	53 (64.6)	54 (67.5)	0.827
Married/living together	29 (35.4)	26 (32.5)	
**BMI ^b^**			
Underweight/normal (<25)	34 (28.1)	28 (23.3)	0.485
Overweight/obese (≥125)	87 (71.9)	92 (76.7)	
**Insurance ^b^**			
Private Insurance	38 (39.2)	38 (38.4)	0.331
Government Health Insurance Plan (Vital)	34 (35.1)	26 (26.3)	
Other/none	25 (25.8)	34 (34.3)	
**Health regions ^b^**			
Aguadilla/Mayagüez	1 (0.9)	4 (3.8)	0.199
Arecibo	11 (10.3)	12 (11.3)	
Bayamón	21 (19.6)	24 (22.6)	
Caguas	21 (19.6)	23 (21.7)	
Fajardo	1 (0.9)	5 (4.7)	
Metro	48 (44.9)	31 (29.2)	
Ponce	4 (3.7)	7 (6.6)	
**Current smoker ^b^**			
No	14 (11.6)	8 (7.1)	0.341
Yes	107 (88.4)	105 (92.9)	
**Current drinker** ^b^			
No	42 (34.4)	29 (25.9)	0.202
Yes	80 (65.6)	83 (74.1)	
**Diagnosed with diabetes mellitus** ^b^			
Yes	100 (83.3)	85 (72.0)	0.0525
No	20 (16.7)	33 (28.0)	
**Family member diagnosed with CRC ^b^**			
Yes	83 (70.9)	92 (78.0)	0.278
No	34 (29.1)	26 (22.0)	
**Location ^b^**			
Proximal	0 (0.0)	20 (17.4)	N/A
Distal	0 (0.0)	91 (79.1)	N/A
Colon unclassified	0 (0.0)	4 (3.5)	N/A
**CRC stages ^b^**			
I	0 (0.0)	26 (21.0)	N/A
II	0 (0.0)	39 (31.5)	N/A
III	0 (0.0)	49 (39.5)	N/A
IV	0 (0.0)	10 (8.1)	N/A
**Dichotomized stages ^b^**			
Early	0 (0.0)	65 (52.4)	N/A
Advanced	0 (0.0)	59 (47.6)	N/A
**Ancestry ^a^**			
African	0.21 (0.14)	0.21 (0.10)	0.9183
Amerindian	0.18 (0.07)	0.19 (0.07)	0.0844
European	0.62 (0.14)	0.60 (0.12)	0.4114
***mSEPT9* ^b^**			
Negative	71 (59.2)	41 (33.3)	0.0001
Positive	49 (40.8)	82 (66.7)	

^a^ Mann–Whitney–Wilcoxon test; ^b^ Pearson chi-square test.

**Table 2:** Descriptive statistics by biomarkers for CRC and healthy controls.

**Overall**							
**Biomarker**	**Control**			**CRC**			***p*-Value ^d^**
	***n***	**Mean ± sd (pg/mL) ^a^**	**Median [Min–Max]** **(pg/mL) ^a^**	***n***	**Mean ± sd** **(pg/mL) ^a^**	**Median [Min–Max]** **(pg/mL) ^a^**	
*mSEPT9* ^b^	120	0.4 ± 0.5	0 [0–1]	123	0.7 ± 0.5	1 [0–1]	<0.001
IGFBP2 ^c^	124	77,686.9 ± 112,386.3	59,483.9 [10,669.3–1,150,318.5]	122	108,648.4 ± 94,665.4	82,894 [24,672.8–603,590.3]	<0.001
DKK3 ^c^	124	34,991.4 ± 16,655.7	30,081.9 [7,606.5–122,490.5]	123	39,655 ± 14,491.9	37,660.2 [14,713.2–87,652.1]	0.002
PKM2 ^c^	122	412.2 ± 426.9	264.8 [6.4–2830.8]	118	521.5 ± 1440.1	260 [7.8–15,226.9]	0.839
**Early**							
**Biomarker**	**Control**			**CRC**			***p*-Value ^d^**
	***n***	**Mean ± sd** **(pg/mL) ^a^**	**Median [Min–Max]** **(pg/mL) ^a^**	***n***	**Mean ± sd** **(pg/mL) ^a^**	**Median [Min–Max]** **(pg/mL) ^a^**	
*mSEPT9* ^b^	63	0.4 ± 0.5	0 [0–1]	65	0.7 ± 0.5	1 [0–1]	0.002
IGFBP2 ^c^	65	87,462.9 ± 140,240.4	62,499.4 [18,390.1–1,150,318.5]	65	101,994.8 ± 81,791.1	85,617.8 [30,020.4–592,800.1]	0.004
DKK3 ^c^	65	37,486.2 ± 18,180.9	34,392.6 [12,774.1–122,490.5]	65	41,205.2 ± 13,592.5	39,873.7 [16,892.5–87,652.1]	0.032
PKM2 ^c^	64	469.5 ± 518.2	253.1 [6.4–2830.8]	63	714.8 ± 1929.8	340.1 [7.8–15,226.9]	0.310
**Advanced**							
**Biomarker**	**Control**			**CRC**			***p*-Value ^d^**
	***n***	**Mean ± sd** **(pg/mL) ^a^**	**Median [Min–Max]** **(pg/mL) ^a^**	***n***	**Mean ± sd** **(pg/mL) ^a^**	**Median [Min–Max]** **(pg/mL) ^a^**	
*mSEPT9* ^b^	57	0.4 ± 0.5	0 [0–1]	58	0.6 ± 0.5	1 [0–1]	0.012
IGFBP2 ^c^	59	66,916.8 ± 69,704.6	48,588.2 [10,669.3–454,458.1]	57	116,235.9 ± 107,739.8	81,277.8 [24,672.8–603,590.3]	<0.001
DKK3 ^c^	59	32,243 ± 14,453.5	29,241.1 [7606.5–91,130.4]	58	37,917.7 ± 15,370.4	35,332.9 [14,713.2–81,936.8]	0.026
PKM2 ^c^	58	349 ± 286.7	282.1 [21.7–1164]	55	300.1 ± 352.5	188.5 [18.6–1787.8]	0.138

^a^ Units apply only to continuous variables; ^b^ *mSEPT9* is a dichotomous variable where 0 was negative, and 1 was positive; ^c^ biomarkers measured as continuous variables; ^d^ Mann–Whitney–Wilcoxon test for medians.

**Table 3:** Comparison of individual biomarkers by early and advanced CRC stages.

Biomarker	Early			Advanced			*p*-Value ^d^
	*n*	Mean ± sd (pg/mL) ^a^	Median [Min–Max] (pg/mL) ^a^	*n*	Mean ± sd (pg/mL) ^a^	Median [Min–Max] (pg/mL) ^a^	
*mSEPT9* ^b^	65	0.7 ± 0.5	1 [0–1]	58	0.6 ± 0.5	1 [0–1]	0.5268
IGFBP2 ^c^	65	101,994.8 ± 81,791.1	85,617.8 [30,020.4–592,800.1]	57	116,235.9 ± 107,739.8	81,277.8 [24,672.8–603,590.3]	0.8414
DKK3 ^c^	65	41,205.2 ± 13,592.5	39,873.7 [16,892.5–87,652.1]	58	37,917.7 ± 15,370.4	35,332.9 [14,713.2–81,936.8]	0.0873
PKM2 ^c^	63	714.8 ± 1929.8	340.1 [7.8–15,226.9]	55	300.1 ± 352.5	188.5 [18.6–1787.8]	0.0014

^a^ Units apply only to continuous variables; ^b^ *mSEPT9* is a dichotomous variable where 0 was negative, and 1 was positive; ^c^ biomarkers measured as continuous variables; ^d^ Mann–Whitney–Wilcoxon test for medians.

**Table 4:** Diagnostic accuracy of *mSEPT9*, IGFBP2, DKK3, and PKM2 overall, early, and advanced stages for CRC detection.

Biomarker	*n*	AUC (95% CI) ^a^	Sensitivity ^b^	Specificity ^b^	PPV ^b^	NPV ^b^
**Overall (*n* = 248)**						
*mSEPT9*	243	62.9 (56.8–69.0)	66.7 (57.6–74.9)	59.2 (49.8–68.0)	62.6 (53.7–70.9)	63.4 (53.8–72.3)
IGFBP2	246	69.7 (63.1–76.3)	67.2 (58.1–75.4)	66.9 (57.9–75.1)	66.7 (57.6–74.9)	67.5 (58.4–75.6)
DKK3	247	61.6 (54.6–68.6)	58.5 (49.3–67.3)	58.1 (48.9–66.9)	58.1 (48.9–66.9)	58.5 (49.3–67.3)
PKM2	240	50.8 (43.4–58.1)	49.2 (39.8–58.5)	49.2 (40.0–58.4)	48.3 (39.1–57.6)	50.0 (40.7–59.3)
**Early (*n* = 130)**						
*mSEPT9*	128	64.0 (55.6–72.3)	69.2 (56.6–80.1)	58.7 (45.6–71.0)	63.4 (51.1–74.5)	64.9 (51.1–77.1)
IGFBP2	130	64.6 (55.0–74.1)	66.2 (53.4–77.4)	61.5 (48.6–73.3)	63.2 (50.7–74.6)	64.5 (51.3–76.3)
DKK3	130	60.9 (51.2–70.7)	64.6 (51.8–76.1)	50.8 (38.1–63.4)	56.8 (44.7–68.2)	58.9 (45.0–71.9)
PKM2	127	55.2 (45.0–65.4)	61.9 (48.8–73.9)	50.0 (37.2–62.8)	54.9 (42.7–66.8)	57.1 (43.2–70.3)
**Advanced (*n* = 118)**						
*mSEPT9*	115	61.7 (52.8–70.7)	63.8 (50.1–76.0)	59.6 (45.8–72.4)	61.7 (48.2–73.9)	61.8 (47.7–74.6)
IGFBP2	116	75.5 (66.6–84.4)	68.4 (54.8–80.1)	72.9 (59.7–83.6)	70.9 (57.1–82.4)	70.5 (57.4–81.5)
DKK3	117	62.0 (51.7–72.2)	51.7 (38.2–65.0)	66.1 (52.6–77.9)	60.0 (45.2–73.6)	58.2 (45.5–70.2)
PKM2	113	58.1 (47.4–68.8)	34.5 (22.2–48.6)	48.3 (35.0–61.8)	38.8 (25.2–53.8)	43.8 (31.4–56.7)

^a^ IGFBP2, DKK3, and PKM2 were analyzed as continuous variables. ^b^ IGFBP2, DKK3, and PKM2 were analyzed as categorical variables using the median as the cut-off. PPV: positive predictive value; NPV: negative predictive value.

**Table 5:** Diagnostic accuracy of biomarkers’ multivariate models overall (a), early (b), and advanced stages (c) for CRC detection.

Biomarker Model	Cut-Off	AUC (95% CI)	Sensitivity	Specificity	PPV	NPV
**Overall (*n* = 248)**						
Model 1 ^a^	0.5178	74.4 (68.1–80.6)	67.8 (58.6–76.1)	67.8 (58.6–76.1)	67.8 (58.6–76.1)	67.8 (58.6–76.1)
Model 2 ^b^	0.4989	74.2 (68.1–80.4)	56.6 (47.3–65.5)	73.3 (64.5–81.0)	68.3 (58.3–77.2)	62.4 (53.9–70.4)
Model 3 ^c^	0.5405	73.5 (67.4–79.5)	42.6 (33.7–51.9)	87.5 (80.2–92.8)	77.6 (65.8–86.9)	60.0 (52.3–67.3)
**Early (*n* = 130)**						
Model 1 ^a^	0.4787	73.7 (65.0–82.4)	61.9 (48.8–73.9)	71.0 (58.1–81.8)	68.4 (54.8–80.1)	64.7 (52.2–75.9)
Model 2 ^b^	0.5124	72.3 (63.7–81.0)	56.9 (44.0–69.2)	68.3 (55.3–79.4)	64.9 (51.1–77.1)	60.6 (48.3–72.0)
Model 3 ^c^	0.4924	71.7 (63.2–80.2)	43.1 (30.8–56.0)	85.7 (74.6–93.3)	75.7 (58.8–88.2)	59.3 (48.5–69.5)
**Advanced (*n* = 118)**						
Model 1 ^a^	0.5087	78.6 (70.0–87.1)	63.6 (49.6–76.2)	80.4 (67.6–89.8)	76.1 (61.2–87.4)	69.2 (56.6–80.1)
Model 2 ^b^	0.5520	76.2 (67.6–84.9)	68.4 (54.8–80.1)	71.9 (58.5–83.0)	70.9 (57.1–82.4)	69.5 (56.1–80.8)
Model 3 ^c^	0.5968	75.4 (66.7–84.0	68.4 (54.8–80.1)	71.9 (58.5–83.0)	70.9 (57.1–82.4)	69.5 (56.1–80.8)

^a^ Model 1: m*SEPT9* + IGFBP2 + DKK3 + PKM2; ^b^ Model 2: *mSEPT9* + IGFBP2 + DKK3; ^c^ Model 3: *mSEPT9* + IGFBP2. Variables IGFBP2, DKK3, and PKM2 are categorical values using the median as the cut-off. PPV: positive predictive value; NPV: negative predictive value.

**Table 6:** Delong test-based ROC comparison of multivariate and univariate models.

Models to Compare	Overall		Early Stage		Advanced Stage	
	AUC (%)	*p*-Value	AUC (%)	*p*-Value	AUC (%)	*p*-Value
**Multivariate Model Comparisons**						
Model 1 ^a^ vs. Model 2 ^b^	74.4	0.9775	73.7	0.8328	78.6	0.7069
	74.2		72.3		76.2	
Model 1 vs. Model 3 ^c^	74.4	0.8392	73.7	0.7514	78.6	0.6047
	73.5		71.7		75.4	
Model 2 vs. Model 3	74.2	0.2969	72.3	0.6756	76.2	0.4017
	73.5		71.7		75.4	
**Model 1 vs. Univariate Models**						
Model 1	74.4	-	73.7	-	78.6	-
*mSEPT9*	62.9	<0.05	64.0	0.116	61.7	<0.05
IGFBP2	69.7	0.3166	64.6	0.1685	75.5	0.6239
DKK3	61.6	<0.05	60.9	0.0579	62.0	<0.05
PKM2	50.8	<0.05	55.2	<0.05	58.1	<0.05

^a^ Model 1: *mSEPT9* + IGFBP2 + DKK3 + PKM2; ^b^ Model 2: *mSEPT9* + IGFBP2 + DKK3; ^c^ Model 3: *mSEPT9* + IGFBP2.

## Data Availability

Data supporting the findings of this study are available from the authors upon request from the corresponding author.
